# Crystallography of Contemporary Contact Insecticides

**DOI:** 10.3390/insects13030292

**Published:** 2022-03-15

**Authors:** Bryan Erriah, Xiaolong Zhu, Chunhua T. Hu, Bart E. Kahr, Alexander Shtukenberg, Michael D. Ward

**Affiliations:** Department of Chemistry and Molecular Design Institute, New York University, New York, NY 10003, USA; be733@nyu.edu (B.E.); xz1668@nyu.edu (X.Z.); chunhua.hu@nyu.edu (C.T.H.); as5243@nyu.edu (A.S.)

**Keywords:** deltamethrin, imidacloprid, bifenthrin, β-cyfluthrin, etofenprox, α-cypermethrin, λ-cyhalothrin, thiacloprid, malaria, mosquitoes

## Abstract

**Simple Summary:**

The efficacy of crystalline contact insecticides is dependent foremost on the uptake of insecticide molecules by insect tarsi contacting crystal surfaces. Insecticide molecules, however, may organize in more than one way in the crystalline state, resulting in more than one crystalline form (also known as *polymorph*). We recently discovered that the lethality of contact insecticides increases with decreasing thermodynamic stability of the crystalline forms; the most stable crystalline form is invariably the least lethal/slowest acting. Polymorphism in contact insecticides, and its importance to efficacy, was largely unknown to the vector control community. It is argued that the crystallographic characterization of contact insecticide solids should be systematic to identify more active solid forms. Herein, we report seven new crystal structures, mostly pyrethroid insecticides recommended by the WHO for indoor residual spraying, as well as a new form of a neonicotinoid insecticide. These results further highlight polymorphism in contact insecticides and the importance of solid-state chemistry in the search for more active crystal forms.

**Abstract:**

The active forms of contact insecticides used for combatting mosquito-borne infectious diseases are typically crystalline solids. Numerous molecular crystals are polymorphic, crystallizing in several solid forms characterized by different physicochemical properties, including bioavailability. Our laboratory recently found that the activity of crystalline contact insecticides is inversely dependent on the thermodynamic stability of their polymorphs, suggesting that efficacy can be enhanced by the manipulation of the solid-state structure. This paper argues that crystallography should be central to the development of contact insecticides, particularly because their efficacy continues to be compromised by insecticide resistance, especially among *Anopheles* mosquito populations that spread malaria. Although insecticidal compounds with new modes of action have been introduced to overcome resistance, new insecticides are expensive to develop and implement. The repurposing of existing chemical agents in metastable, more active crystalline forms provides an inexpensive and efficient method for ‘evergreening’ compounds whose risks are already well-established. We report herein seven new single-crystal structures of insecticides used for controlling infectious disease vectors. The structures reported herein include pyrethroid insecticides recommended by the WHO for indoor residual spraying (IRS)-bifenthrin, β-cyfluthrin, etofenprox, α-cypermethrin, and λ-cyhalothrin as well as the neonicotinoid insecticide thiacloprid.

## 1. Introduction

Contact insecticides are often crystalline. These ingredients function when insect tarsi touch particle surfaces, leading the fight against malaria and other vector-borne diseases. Whilst much effort has been expended in the development of new compounds with improved efficacy, little attention has been paid to the solid-state structure of crystals that the insects encounter. Recent reports from our laboratory have revealed that the insecticidal activity of a particular contact poison depends on the crystal structures and associated free energies of its solid forms, also known as polymorphs, which often are accessible under ambient conditions [1,2,3,4,5]. Some crystal forms of a given contact insecticide can knock down mosquitoes twelve times faster than the commercially available form [1,2]

The efficacy of insecticides is diminished by the development of resistance, which threatens the substantial progress made against malaria in this century [6,7,8,9]. The rapid uptake of an insecticide by insect tarsi upon contact with crystal surfaces is essential for overwhelming insecticide resistance, a consequence of various detoxification reactions [10,11]. If the rate of insecticide uptake can be increased, the toxicant may overwhelm resistance mechanisms. Whilst new insecticides, repellents, and anti-malarial compounds have been introduced in recent years, the introduction of new chemical agents in the field requires sizeable investments of labor and capital [12,13,14,15]. Consequently, the repurposing of existing chemical compounds through manipulation of their crystal structure can be faster, less expensive, and less risky because new compositions of matter are obviated [16].

Polymorphism, a common property of molecular solids [17], is the existence of two or more solid crystalline phases of the same compound. Weak intermolecular interactions and associated shallow potential energy hypersurfaces readily lead to solid forms with a different molecular organization in the solid state, accompanied by distinct chemical and physical properties among the different forms. The presentation of molecules at the crystal surfaces will differ among a family of polymorphs, leading to differences in the chemical potential of molecules at the surface. This is expected for each symmetry-independent facet of a given polymorph as well. The ease of cuticular extraction of insecticide molecules from crystal surfaces would be expected to increase with the increasing chemical potential of the crystal surfaces.

We demonstrated previously that metastable forms of insecticides such as DDT (dichlorodiphenyltrichloroethane), lindane, and fluorinated DDT congeners have greater activity than their most thermodynamically stable polymorphs [3,4,5]. A second DDT polymorph (Form II), first identified by McCrone [18], was characterized and found to be more active than Form I against *Drosophila melanogaster* [3].The inverse correlation between lethality and thermodynamic stability of polymorphs was demonstrated further by two newly characterized polymorphs of lindane, Forms II and III. Knockdown measurements for lindane Forms I, II, and III against *Drosophila melanogaster* revealed that the least stable polymorph kills twice as fast as the commercial Form I [4]. We also discovered a new crystalline form of the difluoro congener of DDT, DFDT (1,1′-(2,2,2-trichloroethane-1,1-diyl)bis(4-fluorobenzene)), as well as its amorphous form. The amorphous form (the least thermodynamically stable solid) was approximately three times faster acting than the thermodynamically stable form (Form I) towards *Anopheles quadrimaculatus* [5]. Moreover, chiral MFDT (1,1,1-trichloro-2,2-(4-chlorophenyl)-(4-fluorophenyl)ethane), a monofluorinated congener of DDT, also exhibited the inverse correlation between crystal thermodynamic stability and insecticidal activity.

**Figure 1:**
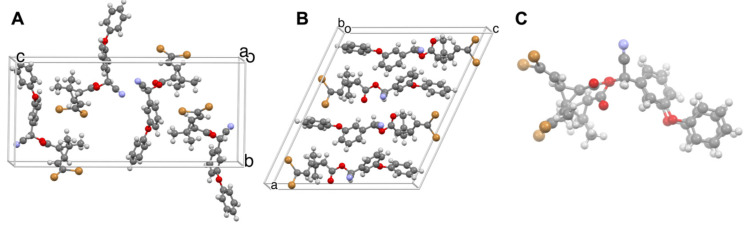
(**A**) Single-crystal structure of DM Form I. (**B**) Single-crystal structure of DM Form II. (**C**) The conformations of DM in Forms I and II overlaid, illustrating distinct molecular conformations in the polymorphs. The crystal structure of Form II was reported for the first time by our laboratory [1]. The crystal structure of DM Form I, redetermined by our laboratory is identical to that previously reported [19].

We also observed identical trends for newly discovered polymorphs of deltamethrin (DM) and imidacloprid (IMI) [1,2], among the most widely used insecticides today. A second DM polymorph, denoted Form II after structural characterization by our laboratory [1], was found to be nine and twelve times faster acting than Form I against *Aedes aegypti* and *Anopheles quadrimaculatus* mosquitoes, respectively [1]. The two polymorphs not only differ with respect to the molecular arrangement in the solid state and the molecular presentations at their crystal surfaces, but they also differ with respect to the conformation of the DM molecules in the crystal lattice (Figure 1). Subsequently, we discovered new polymorphs of imidacloprid with different molecular conformations [2], the least stable polymorph exhibiting nine times greater activity against these mosquitoes than the commercial thermodynamically stable form. Importantly, these metastable forms were found to be stable against transformation to the thermodynamically stable form for at least six months, meeting World Health Organization guidelines for practical use in the field.

The role of polymorphism in contact insecticide formulations has largely been unrecognized by the vector control community. The observation that insect mortality is correlated directly with crystal free energy (or inversely with crystal thermodynamic stability) was not known before our reports, and the observations for so many examples make this link between crystal energy and insecticidal activity statistically robust. Moreover, we have yet to find a compound with multiple polymorphs that is contrary to this trend. Having established a compelling link between crystal polymorphism of contact insecticides and vector control efficacy, we have commenced a comprehensive investigation of polymorphism in contact insecticides, with particular attention to their relative stabilities, both thermodynamic and kinetic. Twelve compounds have been recommended for indoor residual spraying (IRS) by the WHO [20], nine of which are crystalline at room temperature. Single-crystal structures of bifenthrin (BF), etofenprox (ET) and β-cyfluthrin (β-CF) are reported herein for the first time, as well as three new polymorphs of three other compounds: α-cypermethrin (α-CP), λ-cyhalothrin (λ-CH) and thiacloprid (TC).

**Figure 2:**
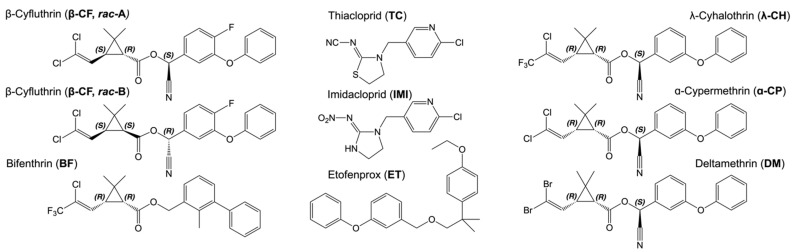
Molecular structures of the insecticides discussed herein. Notes on stereochemistry: Configurations of stereogenic centers are read directly in structures above from left to right throughout, a shortcut past naming conventions that are cumbersome here. β-cyfluthrin (β-CF): *SRS*-enantiomer shown in racemic mixture A (*rac*-A), and the *SSR*-enantiomer in racemic mixture B (*rac*-B). Bifenthrin (BF): *RR*-enantiomer of a racemic mixture is shown. λ-cyhalothrin (λ-CH): *RRS*-enantiomer of a racemic mixture is shown. α-cypermethrin (α-CP): *RRS*-enantiomer of racemic mixture shown. Deltamethrin (DM, *RRS* stereoisomer) is enantiomerically pure.

Certain atoms in the molecular structure of Figure 2 are labeled (*R*) or (*S*), which is the convention for distinguishing the arrangements of chemical groups attached to a so-called stereogenic atom [21]. DM has three such stereogenic atoms. Each such atom can give rise to two stereoisomers. The number of stereoisomers is 2*^N^*, where *N* is the number of stereogenic centers. Consequently, 2^3^ = 8 for DM. Organic synthesis frequently gives rise to a mixture of stereoisomers. Each would have a unique crystal structure. Racemic compounds often contain enantiomeric pairs in crystals. One stereoisomer or racemate can still be polymorphic, however. Deltamethrin, because of some fortuitous aspects of its synthesis, is generated only as the *RRS* stereoisomer (Figure 2) [7]. Because of the flexibility around the eight single bonds (C–C, and C–O), even a single, stable stereoisomeric configuration can lead to multiple polymorphs as illustrated in Figure 1C for DM, a superposition of the (*RRS*) Forms I and II.

## 2. Materials and Methods

*Bifenthrin* (BF, CAS Number 82657-04-3) was purchased from Dr. Ehrenstorfer GmbH (Augsburg, Germany) and used as supplied. BF was grown by lowering the temperature of a supersaturated solution of ethyl acetate from 50 °C to 4 °C, the solution was kept at 4 °C until crystals were seen, at which point it was allowed to stand at room temperature.

*β-Cyfluthrin* (β-CF, a solid mixture comprising the racemate *RSS/SRR* (β-CF, *rac-*A) in 2:1 ratio with the diastereomeric crystal racemate *RSR/SRS* (β-CF, *rac-*B), CAS Number 1820573-27-0, was purchased from Sigma Aldrich (St. Louis, MO, USA). β-CF *rac-*A was grown by slow evaporation from a saturated methanol solution at room temperature. β-CF *rac-*A and *rac-*B were grown from mineral oil at 4 °C.

*Etofenprox* (ET, CAS Number 80844-07-1) was purchased from Sigma Aldrich (St. Louis, MO, USA). A single crystal of ET was retrieved directly from the bottle purchased from the manufacturer (Sigma Aldrich, St. Louis, MO, USA).

*α-Cypermethrin* (α-CP, CAS 67375-30-8) was purchased from Sigma Aldrich (St. Louis, MO, USA). A single crystal of α-CP was grown by cooling its melt to 75 °C on a glass slide mounted on a microscope hot stage (Mettler FP82HT) at 75 °C.

*λ-Cyhalothrin* (λ-CH, CAS Number 91465-08-6) was purchased from Sigma Aldrich (St. Louis, MO, USA). Crystals of λ-CH Form I were grown from the melt at room temperature. The melt of form I was seeded with α-CP to yield λ-CH Form II, which then grew from the melt at room temperature (Figure S1).

*Thiacloprid* (TC, CAS Number 111988-49-9) TC was purchased from Sigma Aldrich (St. Louis, MO, USA). Forms I and II of thiacloprid were grown at room temperature by slow evaporation from saturated solutions of acetone and ethyl acetate, respectively.

All solvents were purchased from Sigma Aldrich (St. Louis, MO, USA) and used as supplied. Complete descriptions of single-crystal X-ray structure analysis, powder diffraction, and spectroscopic characterization are available in the accompanying Supporting Information.

## 3. Results and Discussion

**Figure 3:**
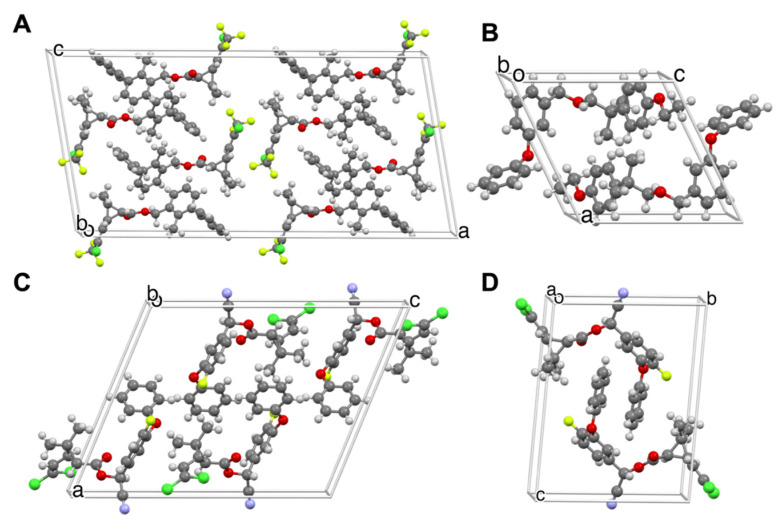
Crystal structures of (**A**) bifenthrin (BF), (**B**) etofenprox (ET), (**C**) *rac-*A β-cyfluthrin (β-CF), and (**D**) *rac-*B β-cyfluthrin (β-CF).

Bifenthrin (BF) (Figure 3A) is used against malaria and filaria vectors. It has been established that the (*RR*)-stereoisomer is 300 times more active against insects than (*SS*), which is 3–4 times more toxic to humans [22]. Crystals of a racemic mixture of BF (*RR* and *SS* stereoisomers) were grown by evaporation of an ethyl acetate solution. The crystal structure was determined at 200 K: monoclinic space group *C*2/*c*, *Z* = 8, *Z’* = 2 (see Table 1). Morphologies of crystals are shown in Figure S3.

The commercially purchased form of β-cyfluthrin (β-CF), a common household insecticide, exists as a mixture comprising *rac-*A and *rac-*B (*RRS* and *SSR*) in a 2:1 ratio, respectively. Block-shaped crystals of β-CF, *rac-*A (Figure 3C), were grown from the commercial mixture by evaporation of a methanol solution in the centrosymmetric monoclinic space group *P2*_1_*/c*, *Z* = 4, *Z’* = 1. β-CF *rac*-B (Figure 3D) was crystallized as {001} needles from a mineral oil solution stored at 4 °C in the triclinic space group $P\bar{1}$, *Z* = 2, *Z’* = 1.

Etofenprox (ET) (Figure 3B) is used to combat malaria and Zika vectors. A single crystal was selected from the manufacturer’s (Sigma Aldrich, St. Louis, MO, USA) bottle and the structure was determined at 100 K. Achiral ET crystallized as {100} plates in the centrosymmetric triclinic space group $P\bar{1}$, *Z* = 2, *Z’* = 1 (Table 1).

**Figure 4:**
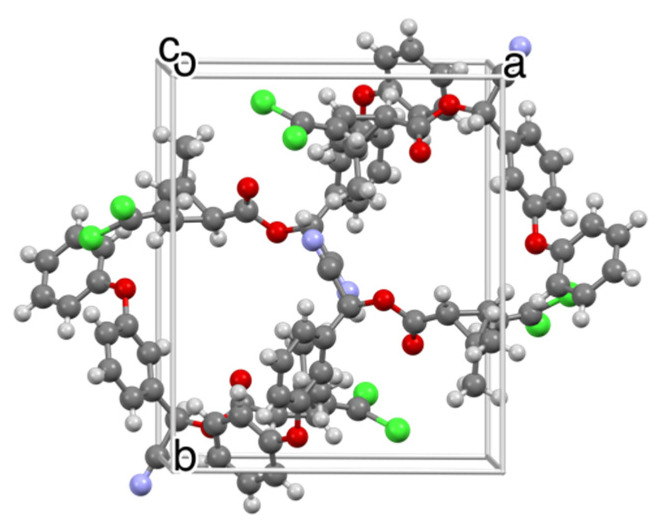
Crystal structure of α-cypermethrin Form I.

A racemic mixture of *cis-*(*RRS*/*SSR*) α-cypermethrin (α-CP) is used in long-lasting insecticide nets and IRS formulations. Five entries appear in the Cambridge Structures Database (CNPOVN, LENDEN, LENDIR, LENDOX, SISYUO), but only CNPOVN and SISYUO contain complete structures [23,24]. Entry CNPOVN (space group *P*$\bar{1}$) is a racemate of the *trans*-(*RRR/SSS*) isomers and SISYUO (space group *P*2_1_2_1_2_1_) is the *cis*-*RRS* isomer. Plates of α-cypermethrin were obtained by cooling the melt. They were refined in the centrosymmetric monoclinic space group *P2*_1_*/n*, *Z* = 4, *Z’* = 1. Consequently, the crystals obtained from the melt correspond to a new polymorph, and the first crystal structure of the commercial form, the enantiomeric pair *cis*-(*RRS/SSR)*, Form I (Figure 4).

**Figure 5:**
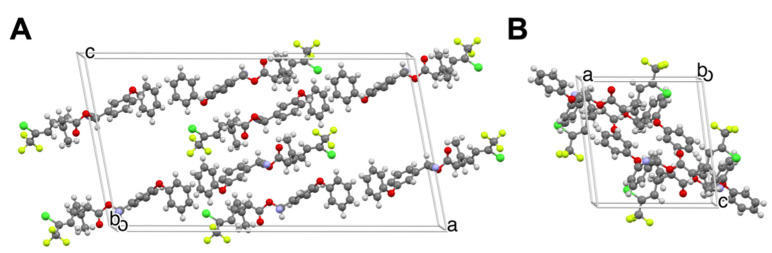
(**A**,**B**) Single-crystal structures of λ-cyhalothrin Forms I (**A**) and II (**B**). In (**A**), the unit cell contains 8 symmetry-related molecules. In (**B**), the unit cell contains 4 symmetry-related molecules.

Cyhalothrin (CH) is a type II pyrethroid with eight possible stereoisomers. The (*SSR*) and (*RRS*) are designations for the stereoisomers of the racemic pair, which comprise a mixture known as λ-cyhalothrin (λ-CH). A structure of λ-CH was reported previously [25]. The mixture of stereoisomers, however, crystallizes as two platy forms. Form I (Figure 5A) was obtained from the melt and crystallized in the centrosymmetric monoclinic space group *C2/c*, *Z* = 8, *Z’* = 1 (See Table 1). Form II (Figure 5B), grown by seeding the melt with α-CP, also crystallizes in a centrosymmetric monoclinic space group, *P*2_1_/*n*, *Z* = 4, *Z’* = 1, (See Table 1).

**Figure 6:**
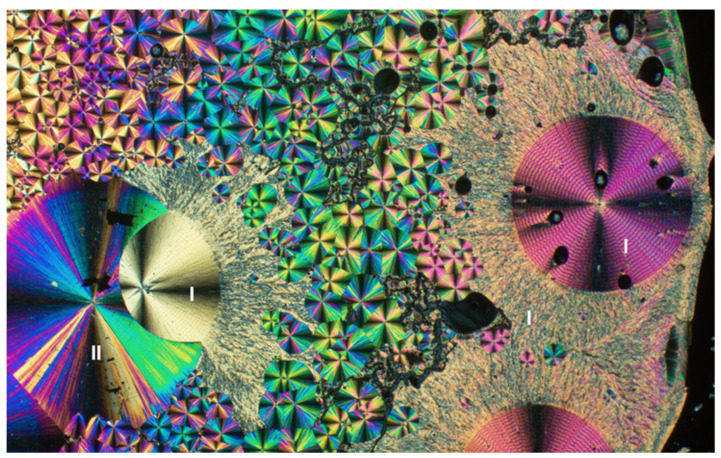
Thin film of thiacloprid crystals grown by cooling its melt, as viewed between crossed polarizers. Form I presents as banded spherulites and a chaotic texture, Form II as smooth spherulites.

**Figure 7:**
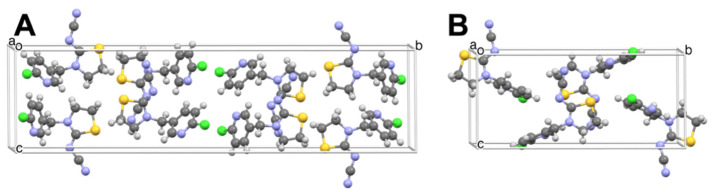
Single-crystal structures of thiacloprid Forms I (**A**) and II (**B**). The unit cell of Form I contains 8 symmetry-related molecules, whereas the unit cell of Form II contains 4 symmetry-related molecules.

Thiacloprid, TC, is a neonicotinoid insecticide like IMI but less toxic to mammals as well as honeybees [26,27]. TC crystallized from the melt as three distinct morphologies: banded spherulites, smooth spherulites, and regions with chaotic texture (Figure 6). The two spherulite morphologies were distinct by Raman microscopy (Figure S2). The chaotic texture and banded spherulites (*T_m_* = 135 ℃) corresponded to the commercially available form, designated Form I, whilst the smooth spherulites (*T_m_* = 125 ℃) corresponded to a new Form II. Form I (Figure 6 and Figure 7A) crystallized as blocks in the monoclinic space group *P*2_1_/*c*, *Z* = 4, *Z*’ = 1 (See Table 1). The structure of Form II (Figure 6 and Figure 7B), grown as needles, was reported previously, and confirmed here as the monoclinic space group *P*2_1_/*c*, *Z* = 8, *Z*’ = 2 [28]. The concentric bands, a consequence of helicoidal twisting of crystallites growing radially, is a common phenomenon among melt-grown molecular crystals, which we have documented thoroughly [29,30,31,32,33].

**Table 1:** Single-crystal X-ray crystallography data obtained for insecticides, and corresponding experimental conditions.

Compound	Fenthrin	Β-Cyfluthrin, *rac-*A	Β-Cyfluthrin, *rac-*B	Etofenprox	α-Cypermethrin	λ–Cyhalothrin		Thiacloprid	
Polymorph	I	I	I	I	I	I	II	I	II
CCDC No.	2142944	2142946	2142945	2142943	2142947	2142941	2142942	2142940	2142939
Formula	C_23_H_22_ClF_3_O_2_	C_22_H_18_Cl_2_FNO_3_		C_25_H_28_O_3_	C_22_H_19_Cl_2_NO_3_	C_23_H_19_ClF_3_NO_3_		C_10_H_9_ClN_4_S	
*M_w_*, g/mol	422.87	434.27		376.50	416.30	449.85		252.72	
Space Group	*C*2/*c*	*P*2_1_/*c*	$P\bar{1}$	$P\bar{1}$	*P*2_1_*/n*	*C*2/*c*	*P*2_1_/*n*	*P*2_1_/*c*	*P*2_1_/*c*
*Z, Z’*	8, 2	4, 1	2, 1	2, 1	4, 1	8,1	4,1	4, 1	8, 2
*a*, Å	35.061 (3)	15.4332 (8)	6.5099 (16)	10.3004 (8)	11.497 (2)	34.273 (2)	11.8222 (9)	7.4438 (14)	7.0305 (3)
*b*, Å	7.1704 (5)	7.5413 (4)	11.086 (3)	10.5102 (8)	13.712 (2)	6.9368 (5)	14.3037 (11)	18.305 (3)	35.2105 (13)
*c*, Å	17.1168 (12)	19.3706 (10)	14.333 (3)	10.6408 (8)	12.972 (2)	18.3172 (12)	12.5427 (10)	8.2436 (15)	9.0164 (3)
*α*, °	90	90	94.487 (10)	86.176 (3)	90	90	90	90	90
*β*, °	99.999 (3)	112.348 (2)	96.984 (11)	63.403 (3)	98.349 (2)	101.2360 (10)	97.1020 (10)	95.439 (6)	98.2269 (11)
*ɣ*, °	90	90	99.455 (11)	87.263 (3)	90	90	90	90	90
*V*, Å^3^	4237.8 (5)	2085.14 (19)	1007.7 (4)	1027.56 (14)	2023.2 (6)	4271.3 (5)	2104.7 (3)	1118.21 (4)	2209.02 (14)
*D*_c_, g/cm^3^	1.326	1.383	1.431	1.217	1.367	1.399	1.420	1.501	1.520
μ, mm^−1^	0.222	0.343	0.355	0.078	0.344	0.230	0.233	0.504	0.510
2*θ* range, °	2.36–28.30	2.18–28.33	1.87–26.97	1.94–28.32	2.174–28.317	1.211–28.288	2.169–28.316	2.23–26.00	2.31–28.30
*T*, K	200	201	295	100	100	100	100	201	200
Total Reflections	5187	5176	4015	5085	5045	5297	5246	2714	5478
Observed Reflections	2595	3318	1642	3319	4452	4208	43842	2811	4586
No. Parameters	265	264	264	256	255	282	282	145	289
*R*_1_[*I* > 2σ(*I*)]	0.0735	0.0630	0.1579	0.0608	0.0349	0.0463	0.0502	0.0369	0.0401
*wR*_2_ all data	0.2601	0.1731	0.4197	0.1506	0.0945	0.1276	0.1354	0.0946	0.1052
GoF	1.033	1.054	1.043	1.022	1.067	1.022	1.007	1.087	1.032

*M_w_* = Molecular Mass, *D*_c_ = Crystallographic Density, μ = Absorption coefficient, GoF = Goodness of fit. Thiacloprid Form II and λ-Cyhalothrin Form I were previously reported [25,28].

## 4. Conclusions

DM, first synthesized in 1973, was the most potent synthetic insecticide ever at the time and was heralded for its high selectivity to insects compared with mammals, a ratio of 13,000 [7]. Upon stereoselective synthesis, the solution contains the (*RRR*) and (*RRS*) stereoisomers (Figure 2). The latter crystallizes more readily, leaving the more soluble (*RRR*) isomer in solution, which can be epimerized at the cyano-bearing carbon atom to produce more *(RRS*). This is fortuitous in that (*RRS*) is the most active insecticide. Stereoisomerism is an essential feature of biological specificity.

More commonly, insecticides are supplied as mixtures of stereoisomers, which can greatly increase the complexity of crystallographic characterizations and give rise to variable crystallization outcomes (See Table S1 for crystallization conditions). The activity of crystalline contact insecticide is dictated foremost by the rate of absorption at the interface between the crystal and the target organism. Yet little attention has been paid to insecticide crystallography. This knowledge gap is exemplified by the twelve insecticides recommended for IRS by the WHO, five of which were not previously characterized crystallographically. Herein, we have reported the characterization of seven new crystallographic forms of six contact insecticides, expanding a comparatively small structural knowledge base. As mentioned above, our laboratory has demonstrated a convincing correlation between contact insecticide activity and the respective free energies of their crystal polymorphs. This behavior remains to be validated for the new characterized materials described herein.

## Data Availability

Crystal structure data reported in this paper have been deposited at the Cambridge Crystallographic Data Centre with accession numbers found in Table 1.

## Supplementary Materials

The following are available online at https://www.mdpi.com/article/10.3390/insects13030292/s1, Table S1: Preparation of insecticide crystals from solution crystallization, Figure S1: Heterogenous nucleation of λ-Cyhalothrin Form II on α-Cypermethrin crystals, Figure S2: Raman spectra of thiacloprid Form I and Form II, Figure S3: Single-crystal images with Miller indices.
