# Supplementary material for: Crystallography of Contemporary Contact Insecticides
Source: Insects. 2022 Mar 15;13(3):292. doi: 10.3390/insects13030292 (PMC8949367; doi:10.3390/insects13030292)
Supplement: Supplementary file 1 [file insects-13-00292-s001.zip › insects-1604670-supplementary.pdf]

Supplementary Information

Crystallography of Contemporary Contact Insecticides

Bryan Erriah,[1] Xiaolong Zhu,[1] Chunhua T. Hu,[1,] Bart Kahr,[1*] Alexander G. Shtukenberg,[1] Michael D. Ward[1*]

[1]Department of Chemistry and Molecular Design Institute, New York University, New York, NY 10003 USA

Corresponding authors: Michael D. Ward; Bart Kahr
Emails: mdw3@nyu.edu (M.D.W.); bart.kahr@nyu.edu (B.K.)

Table of Contents

Materials and Methods

Supplementary Table S1. Preparation of insecticide crystals from solution crystallization.

Supplementary Figure S1. Heterogenous nucleation of λ-cyhalothrin Form II single crystals.

Supplementary Figure S2. Raman spectra of thiacloprid polymorphs.

Supplementary Figure S3. Single crystals with Miller indices.

Crystallographic Information File:

Bifenthrin Form I.cif

β-Cyfluthrin Rac-A.cif

β-Cyfluthrin Rac-B.cif

Etofenprox Form I.cif

α-Cypermethrin Form I.cif

λ-Cyhalothrin Form I.cif

λ-Cyhalothrin Form II.cif

Thiacloprid Form I.cif

Thiacloprid Form II.cif

CheckCIF: 9 structures_checkcif.pdf

Materials and Methods

Materials. *Bifenthrin.* (BF, (*rac*-(2-methyl[1,1'-biphenyl]-3-yl)methyl-(1*R*,3*R*)-3-[(1*Z*)-2-chloro-3,3,3-trifluoroprop-1-en-1-yl]-2,2-dimethylcyclopropane-1-carboxylate, CAS 82657-04-3) was purchased from Dr. Ehrenstorfer GmbH (Augsburg, Germany) and used as supplied. BF was grown by lowering the temperature of a supersaturated solution of ethyl acetate from 50°C to 4°C, the solution was kept at 4°C until nucleation was observed, at which point it was left at room temperature. *β-Cyfluthrin.* (*β*-CF, a reaction mixture comprising of the enantiomeric pair *rac*-(*R/S*)-cyano(4-fluoro-3-phenoxyphenyl)-methyl-(1*S*/*R*,3*S*/*R*)-3-(2,2-dichloroethenyl)-2,2-dimethylcyclopropane-1-carboxylate (*β*-CF, *rac*-A) in ratio 2:1, as reported by the University of Hertfordshire Pesticide Properties DataBase, with the diastereomeric racemate *rac*-(*R*/*S*)-cyano(4-fluoro-3-phenoxyphenyl)methyl-(1*S*/*R*,3*R*/*S*)-3-(2,2-dichloroethenyl)-2,2 - dimethylcyclopropane-1-carboxylate (*β*-CF, *rac*-B); CAS Number 1820573-27-0. *β*-CF *rac*-A was grown from a supersaturated methanol solution at room temperature. *β*-CF *rac*-A and *rac*-B were grown from mineral oil at 4°C. *Etofenprox*. (ET, (1-ethoxy-4- [2-methyl-1- [(3 phenoxy phenyl) methoxy] propan-2-yl] benzene; CAS Number 80844-07-1). A single crystal of ET was retrieved directly from the bottle purchased from the manufacturer, Sigma Aldrich (St. Louis, MO, USA). *a-Cypermethrin.* (*a*-CP, (*rac*-(*R*)-cyano(3-phenoxyphenyl)methyl (1S,3S)-3-(2,2-dichloroethenyl)-2,2-dimethylcyclopropane-1-carboxylate, CAS 67375-30-8. A single crystal of *a* -CP was grown from its melt on a glass slide using a microscope hot stage (Mettler FP82HT) at 75°C. *λ-Cyhalothrin.* (*λ*-CH, *rac*-(*R*/*S*)-cyano-3-phenoxybenzyl (1*S*/*R*,3*S*/*R*)-cis-3-[(*Z*)-2-chloro-3,3,3-trifluoropropenyl]-2,2-dimethylcyclopropanecarboxylate; CAS Number 91465-08-6. Crystals of *λ*-CH form I were grown from the melt. The melt of form I seeded with *a*-CP to yield *λ*-CH form II. Both forms were grown at room temperature. *Thiacloprid.* Forms I and II of thiacloprid (TC, (((2*Z*)-3-[(6-Chloro-pyridin-3-yl)methyl]-1,3-thiazolidin-2-ylidene) cyanamide; CAS Number 111988-49-9) were grown by slow evaporation from saturated solutions of acetone and ethyl acetate, respectively. All solvents and compounds, unless states, were purchased from Sigma Aldrich (St. Louis, MO, USA) and used as supplied.

Raman spectroscopy. Raman spectra were recorded on a Raman microscope (DXR, Thermo Fisher Scientific, Waltham, MA) using a 532 nm excitation laser operating at 2 mW, with a 2 cm^{-1} resolution and slit width of 50 μm. The data was analyzed using the Omnic software package.

Polarized light microscopy. A microscope fitted with crossed polarizers (Olympus BX50) and equipped with a digital camera was used to record crystallization. A microscope hot stage (Mettler FP82HT) was used for temperature control.

Single-crystal structure determination. X-ray intensity data for all crystals were recorded on a Bruker D8 APEX-II and III CCD systems with the ω scan method using graphite-monochromated and 0.5 mm MonoCap-collimated Mo-Kα radiation (λ = 0.71073 Å). The temperature was controlled by an Oxford Cryosystems 700+ Cooler. The data sets were processed with the INTEGRATE program of the APEX2 software for reduction and cell refinement. Multi-scan absorption corrections were applied by the SCALE program for the area detector. Both structures were solved by intrinsic phasing methods (SHELXT) and the structure models were completed and refined using the full-matrix least-square methods on F^2 (SHELXL). Non-hydrogen atoms in the structures were refined with anisotropic displacement parameters, and hydrogen atoms on carbons were placed in idealized positions (C-H = 0.95-1.00 Å) and included as riding with $U_{iso(H)}$ = 1.2 or 1.5 $U_{eq(non-H)}$. The selected crystallographic parameters were listed in Table 1. Crystallographic information files (CIFs), including the HKL and RES data, are deposited in the CCDC with accession numbers found in Table 1. Crystallographic information files of these nine structures are supplied as auxiliary files.

Supplementary Table S1. Preparation of Insecticide crystals from solution crystallization.

Solvent	*Bifenthrin*	*β – Cyfluthrin,*	*Etofenprox*	*Thiacloprid*
Methanol	viscous liquid	*rac*-A	-	Form I
Chloroform	viscous liquid	-	-	-
Dichloromethane	viscous liquid	*rac*-A	-	Form I
Toluene	viscous liquid	*rac*-A	viscous liquid	-
Hexane	viscous liquid	*rac*-A	-	Form I
Acetone	viscous liquid	-	viscous liquid	Form I
Xylene	viscous liquid	-	viscous liquid	-
Ethyl Acetate	Form I	-	viscous liquid	Form II
Ethanol	-	-	-	Form I & II
Mineral Oil	-	*rac*-A & *rac*-B	-	-

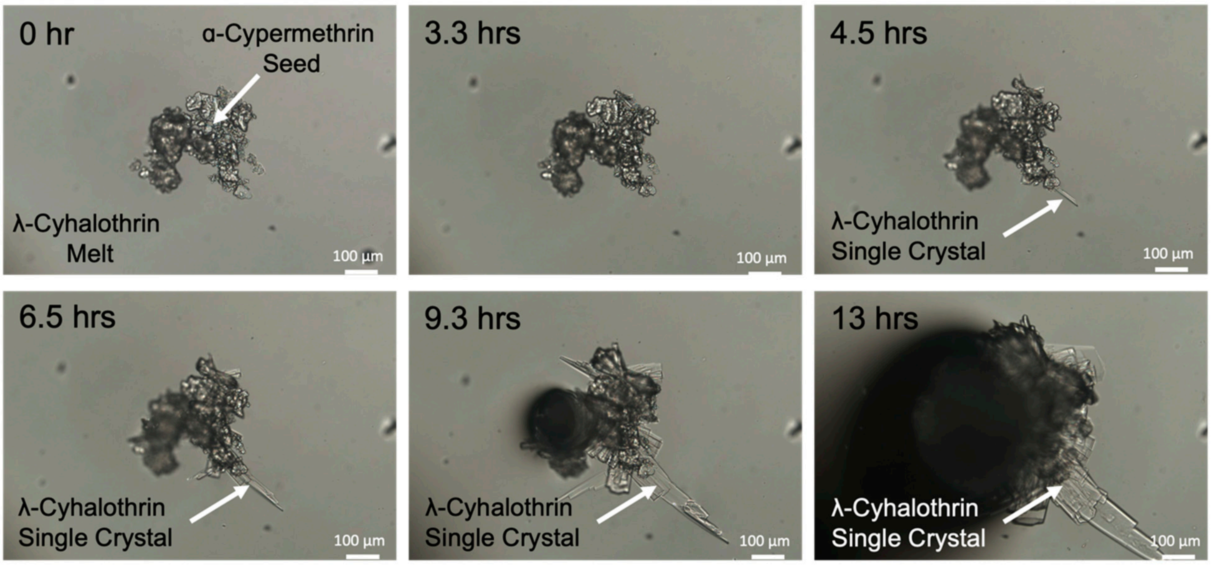

Supplementary Figure S1. Heterogenous nucleation of λ-cyhalothrin Form II on α-cypermethrin crystals: Heterogeneous nucleation of λ-Cyhalothrin Form II was induced by seeding λ-Cyhalothrin melt with commercial α-Cypermethrin single crystals at 75 °C. A polarized light microscope fitted with crossed polarizers (Olympus BX50) and equipped with a digital camera was used to record the crystallization. Note: video not recorded with analyzer in place. A microscope hot stage (Mettler FP82HT) was used for temperature control.

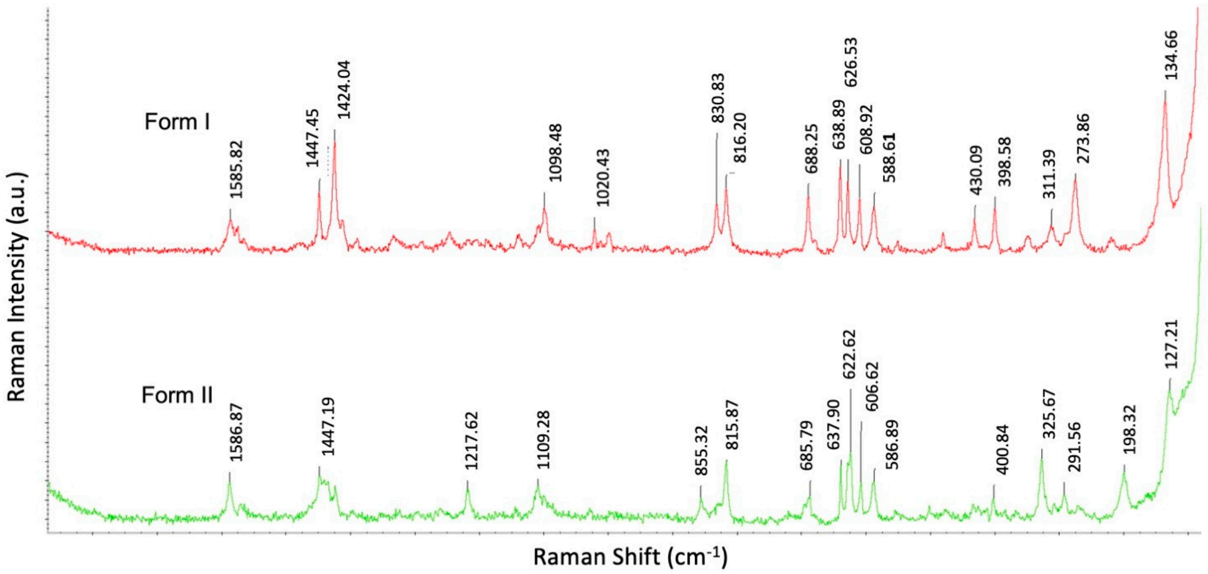

Supplementary Figure S2. Raman spectra of thiacloprid Form I and Form II: Raman spectra were acquired from thin films of each polymorph prepared between glass slides.

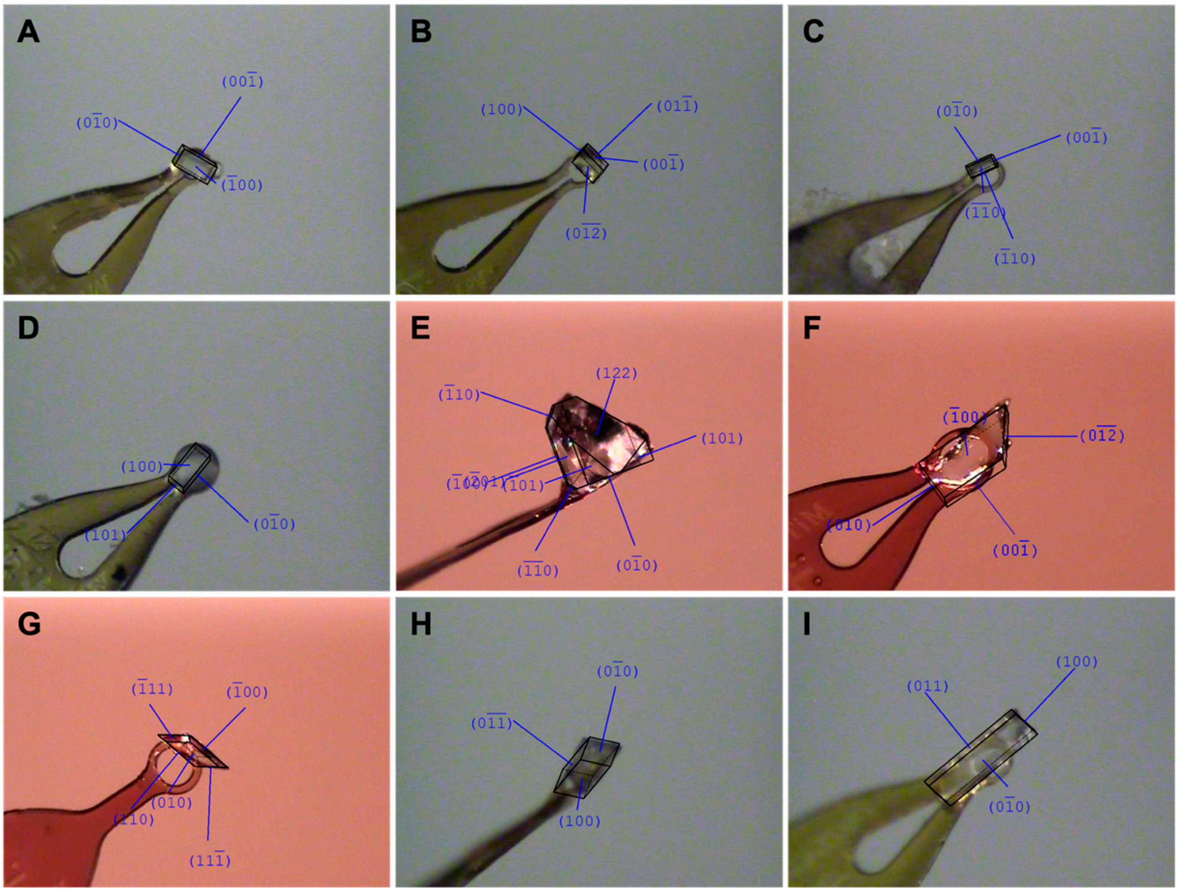

Supplementary Figure S3. Single crystal images with Miller indices: (A) bifenthrin, (B) β-cyfluthrin *rac*-A, (C) *β*-cyfluthrin *rac*-B, (D) etofenprox, (E) α-cypermethrin, (F) λ–cyhalothrin Form I, (G) λ–cyhalothrin Form II, (H) thiacloprid Form I, (I) thiacloprid Form II.
